# The Effect of Consumers and Mutualists of *Vaccinium membranaceum* at Mount St. Helens: Dependence on Successional Context

**DOI:** 10.1371/journal.pone.0026094

**Published:** 2011-10-20

**Authors:** Suann Yang, Eelke Jongejans, Sylvia Yang, John G. Bishop

**Affiliations:** 1 School of Biological Sciences, Washington State University, Pullman, Washington, United States of America; 2 Radboud University Nijmegen, Institute for Water and Wetland Research, Department of Experimental Plant Ecology, Nijmegen, The Netherlands; 3 Department of Biology, University of Washington, Seattle, Washington, United States of America; 4 School of Biological Sciences, Washington State University, Vancouver, Washington, United States of America; University of Hull, United Kingdom

## Abstract

In contrast to secondary succession, studies of terrestrial primary succession largely ignore the role of biotic interactions, other than plant facilitation and competition, despite the expectation that simplified interaction webs and propagule-dependent demographics may amplify the effects of consumers and mutualists. We investigated whether successional context determined the impact of consumers and mutualists by quantifying their effects on reproduction by the shrub *Vaccinium membranaceum* in primary and secondary successional sites at Mount St. Helens (Washington, USA), and used simulations to explore the effects of these interactions on colonization. Species interactions differed substantially between sites, and the combined effect of consumers and mutualists was much more strongly negative for primary successional plants. Because greater local control of propagule pressure is expected to increase successional rates, we evaluated the role of dispersal in the context of these interactions. Our simulations showed that even a small local seed source greatly increases population growth rates, thereby balancing strong consumer pressure. The prevalence of strong negative interactions in the primary successional site is a reminder that successional communities will not exhibit the distribution of interaction strengths characteristic of stable communities, and suggests the potential utility of modeling succession as the consequence of interaction strengths.

## Introduction

The extreme intensity of the disturbance that results in primary succession is generally considered to be responsible for the differences in community assembly between primary and secondary succession. Ecologists have identified a variety of processes whose importance is magnified during primary succession, including amelioration of the physical environment, dispersal limitation, facilitative interactions and stochastic assembly [1], [2], [3], [4], [5], [6], [7], [8], [9]. In contrast, the effect of consumers on successional plant communities is regarded as more important in secondary succession [10], [11] and in marine systems [12], [13], [14], [15]. Similarly, because of their relative scarcity in primary succcesion, mutualists are also thought to be more important in secondary succession, and the ability to grow and reproduce without their aid is considered an important attribute of primary successional plant colonists [8].

Although interactions with consumers and mutualists are considered relatively unimportant for primary succession, a variety of studies indicate that they may strongly affect colonization of plant populations. For example, in early succession, consumers may temporarily escape their enemies and cause unusually large effects on plant population growth and spatial spread [15], [16], [17], [18]. Likewise, the temporary absence of mutualists, such as pollinators (e.g., [19], mycorrhizae [20] and nitrogen-fixing symbionts [e.g.], [ 21,22] may disadvantage or temporarily exclude colonizing plant species that are dependent upon them.

These studies suggest that the limiting effects of biotic interactions on colonizing plants can be greatly amplified during primary succession. This temporary inflation may be caused by successional properties of interaction webs. Primary successional sites, being the most intensely disturbed, generally have few species, low productivity, and support fewer trophic levels [23], whereas secondary successional sites generally possess more complex sets of interacting species. Under these circumstances, consumers may anomalously impact a primary successional plant population, because secondary consumers or competitors that might weaken the interaction are temporarily lacking. These temporarily strong effects of biotic interactions may then translate to higher negative interaction strengths (i.e., effects on population dynamics) in primary successional communities. However, it is not yet known whether the impact of biotic interactions is more frequently promoted in the primary successional context, compared to the secondary successional context. The few existing examples focus on pairwise interactions, while studies of multispecies interaction remain particularly scarce [24], [25]. More systematic examination of ensembles of consumers and mutualists across multiple successional contexts is required.

The success of colonizing plant populations may be influenced by other factors from outside the local community. In particular, propagule pressure may form a key context early in succession, where the shift from immigration from external sites (donor control) to propagule production from within a site (local control) may strongly affect successional rates [15], [26]. In general, the growth and spread of colonizing populations are highly dependent on propagule production and dispersal [e.g.], [ 8], [27,28], and the effects of consumers or mutualists on seed production may be much larger relative to those in stable or declining populations [29], [30].

In this study we asked three questions: 1) Does the limiting effect of multiple species interactions on plant reproduction vary with successional context? 2) Do consumers and mutualists differ in their influence on colonization during primary succession? and 3) How do successional differences in interactions combine with local control of propagule influx to affect colonization during primary succession? To address these questions, we investigated the effect of consumers (a fungal pathogen, pre- and post-dispersal seed predators, and an insect herbivore) and pollinator mutualists on black huckleberry (*Vaccinium membranaceum*) in primary successional and adjacent secondary successional areas created by the 1980 eruption of Mount St. Helens (Washington, USA).

## Materials and Methods

### Study system

**Figure 1:**
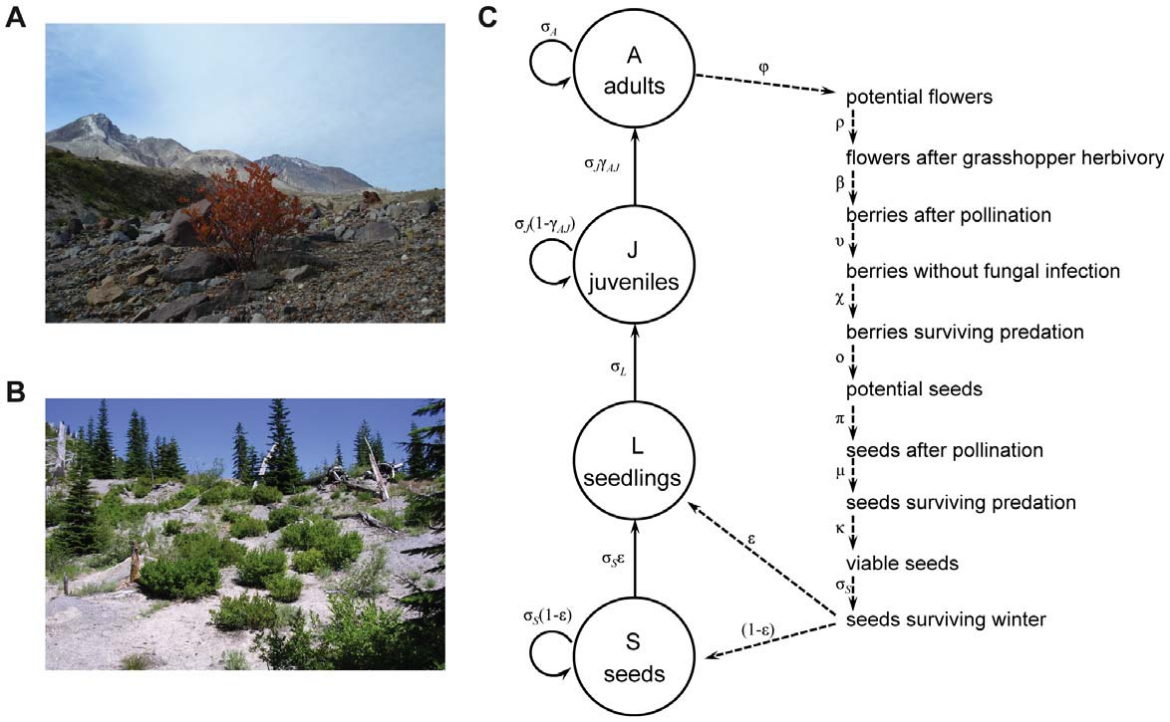
Huckleberry habitat and life cycle diagram. Huckleberry is sparsely distributed in primary successional sites (A), and densely distributed in secondary (B) successional sites. (C) Life cycle diagram for huckleberry. The Greek letters denote the transition rates between the consecutive steps (see Table 1).

*V. membranaceum* (black huckleberry) Dougl. ex Torr. (Ericaceae) is a long-lived, iteroparous perennial shrub that is common in open and forested habitats between 1000–1800 m elevation throughout the Pacific Northwest [31]. Black huckleberry is one of the few animal-dispersed plants beginning to colonize the primary successional Pumice Plain of Mount St. Helens. Mount St. Helens (46°12′N, 122°11′W, Washington, USA) erupted in 1980, creating a 60 km^2^ area of primary successional landscape north of the volcano (including the Pumice Plain) and the surrounding 600 km^2^ of secondary successional habitat [32]. Huckleberry has established on the Pumice Plain at low densities within communities dominated by forbs, graminoids, and mosses, with scattered willows and conifer saplings (Figure 1A; see [33] for vegetation composition). In addition to post-eruption colonists, the black huckleberry population of the Pumice Plain also consists of re-sprouted, surviving individuals in pre-eruption soils (exposed by erosion on a few northeast-facing slopes) [26]. In 2003–2005 we located 21 survivors (surrounded by extensive primary successional habitat as described above) and 68 colonists (none of which were yet reproducing) scattered across the Pumice Plain. Together, these 89 individuals constituted the primary successional population, and have similar aboveground biotic interactions regardless of pre- or post-eruption origin. About 50% of colonists were the offspring of the survivors, with the rest colonizing from secondary successional sources [34]. In nearby secondary successional habitat, where the eruption had a much less intense effect, black huckleberry is abundant (we found 1054.2±81.1 [mean ± SE] individuals/ha), and occurs with other animal-dispersed shrubs and small trees (Figure 1B).

### Consumer and mutualist interactions with huckleberry

We measured the limiting effects of consumers and mutualists on sequential phases of reproduction [35], [36] of huckleberry, i.e., on reproductive rate parameters (as diagrammed in Figure 1C). Reduction in berry (β) and seed (π) production due to lack of pollination, herbivory (ρ), fungal infection (υ), and pre-dispersal seed predation (χ) were each measured on the adults scattered throughout primary successional (PS) and in the nearby (at least 1.5 km distant) secondary successional (SS) huckleberry population. Separate bare-ground plots (as described below) were used to measure post-dispersal seed predation (μ) and seedling survival (σ_L_) for PS and SS. All work was completed under a special use permit from the US Forest Service's Mount St. Helens National Volcanic Monument.

#### Effects of bee pollination on berry (β) and seed (π) production

Huckleberry, being largely self-incompatible, has difficulty reproducing without the services of bee pollinators [37], and the difference in PS and SS plant density may lead to variation in pollination and hence in propagule production. In 2003 and 2004, we quantified the effect of pollination on berry production in PS adults (in soil refugia on the Pumice Plain) and SS adults (located in an area representative of secondary successional huckleberry habitat) as the ratio β = *bnp/bsp*, where *bnp* = berries initiated under natural pollination and *bsp* = berries initiated under supplemental hand pollination. We assumed that this supplementation represents the maximum fruit set possible given resources available at a particular plant location. β<1 indicates that pollinator services are lacking. Supplemented flowers received pollen from several donor plants daily until stigmas were no longer receptive (indicated by a dry stigma and corolla abscission). In 2003 this was done for 5 flowers per treatment on 6 plants in each population (2 treatments×5 flowers×6 plants = 60 flowers); in 2004 it was done for 10 flowers per treatment on 8 plants in each population (2 treatments×10 flowers×8 plants = 160 flowers). We also used the effect of pollination (β) to calculate the number of potential berries (i.e. flowers) per adult (φ), which is defined as the number of berries that would develop if there was no pollinator limitation and no resource limitation to berry production. First, we estimated the total number of berries (*tnb*) on a bush, and then worked backwards to the number of flowers by dividing the number of berries by the effect of pollination ratio: φ = *tnb*/β.

We also collected the mature berries (n = 240) from the 2004 pollination experiment and counted the number of seeds per berry from natural pollination (*snp*) and supplemental hand pollination (*ssp*). The effect of pollination on seed production per berry was then estimated by π = *snp*/*ssp*, with the potential number of seeds per berry being ο = *ssp*.

#### Herbivory (ρ)

During the course of this study (2002–2005), acridid grasshoppers severely defoliated primary successional huckleberry only in 2003 (1 of 4 years), which resulted in a nearly complete loss of fruit production in that year. In contrast, we never observed herbivory of this magnitude on secondary successional huckleberry. We approximated this stark contrast in the rate at which flowers survive herbivory (ρ) with ρ = 1 for SS and in low herbivory years for PS, and with ρ = 0 in a high herbivory year.

#### Fungal infection (υ) and seed predation (χ)

Huckleberry flowers are susceptible to *Monilinia vaccinii-corymbosii* (mummyberry), a fungus that prevents seed formation in *Vaccinium* spp. [38]. In addition, pre-dispersal seed predation by dipteran larvae results in damaged berries that easily desiccate and fall from the plant [37]. To compare the degree of fungal infection and pre-dispersal seed predation in both successional populations, we tracked the fate of 9854 berries on PS survivor adults and SS adults (in an area representative of secondary successional habitat) in weekly censuses in 2004 and 2005. In 2004, we censused a subset of berries (7169 total) on 16 plants in PS (on average 249.8±3.2 s.e. berries per plant) and 18 plants in SS (on average 176.2±4.5 s.e. berries per plant) weekly. After recording the number of berries infected by *Monilinia* and depredated by insects, we removed the affected berries to ensure we did not count them again the following week. This process allowed us to estimate total number of infected (*nib*), depredated (*ndb*), and unaffected (*nub*) berries over the entire season. We repeated weekly counts in 2005, using a total of 2685 berries on ten plants in PS (on average 182.5±9.1 s.e. berries per plant) and on ten in SS (on average 86.0±4.6 s.e. berries per plant). The proportion of berries surviving after infection (υ) and pre-dispersal predation (χ) were estimated as υ = (*nub+ndb*)/(*nub+ndb+nib*) and χ = *nub*/(*nub+ndb*). Note that removing the infected berries is not likely to have a large effect on depredation, and vice versa, due to the phenological mismatch of the two events. During fungal infection, green fruits become mummified as they develop. In contrast, seed predators more often attack fruit after ripening.

#### Post-dispersal seed predation (μ)

Seeds that are still viable in the fecal matter of frugivorous visitors must escape from seed predators, including small mammals and invertebrates such as scarabid beetles. We focused on berries dispersed by coyotes because their berry-filled scats, which can contain over 5,000 seeds each, are the most abundant and frequently encountered compared to the scats of other seed-dispersing frugivores. Birds are also seen consuming huckleberry fruits, but we did not observe droppings with seeds in the primary succession population (PS) as frequently (and each bird dropping carries far fewer seeds than a coyote scat). In addition, we systematically surveyed for seeds or seedlings beneath potential bird perches, and found none. In the absence of information suggesting otherwise, we considered bird dispersal to be rare, and focused on dispersal by coyotes. In 2004 and 2005, we collected fresh coyote scats that contained huckleberry seeds and pulp. In 2004, we placed an average of 8.22±0.10 s.e. grams of scat in each of 12 plots in one primary and one secondary successional location (24 plots total). In 2005, we used 3 locations, at least 200 m apart, in each successional stage, with 6 plots in each location (36 plots total). We also counted the number of seeds in a subsample of fecal material to estimate the density of seeds per scat (*dss*; seeds/g scat) to estimate the total number of seeds (*tns*) placed in each plot: *tns* = g scat × *dss*. We checked for seedling emergence each week for 1 month (no seedlings emerged), after which we counted the remaining number of seeds (*rns*). As we found no seedlings emerging in a 4–5 m radius area around the plots during each weekly check, we refer to post-dispersal seed removal-related mortality as “seed predation,” calculated as μ = (*tns-rns*)/*tns*. Additionally, PS sites were checked in subsequent years, and no seedlings were detected.

#### Seed viability (κ)

To estimate seed viability (κ), we removed seeds from coyote scats, placed them on nutrient-free agar with 1 mL 10^−3^ M gibberellic acid (GA3) solution to break dormancy (Giba et al. 1993), and counted the number of germinable seeds (*ngs*) out of the total seeds tested (*nts*): κ = *ngs/nts* over a 3 week period.

### Analyses

To investigate whether species interactions vary with successional context (Question 1), we analyzed the different species interactions separately and then together. First, we used generalized linear models with binomial or quasipoisson error-distributions in R [39] to test for differences between PS and SS in pollination limitation, fungal infection, and pre- and post-dispersal seed predation. We also estimated the population-level negative effects of these biotic interactions on reproduction (hereafter, reproductive effects), and compared them between primary and secondary succession. We calculated reproductive effect as ln(mean reproductive rate), which is equivalent to the more usual log response ratio calculation ln(N*_s_*_+1_/N*_s_*) [25], [40], where N*_s_* is the number of ovules or seeds before a particular interaction, and N*_s_*_+1_ is the number after that interaction (in the temporal order of herbivory, pollination, fungal infection, pre-dispersal seed predation, and post-dispersal seed predation). This estimate of reproductive effects of consumers and mutualists does not account for variation in interactions among years. Thus, we complemented the log response ratio estimate with transition matrix-based stochastic simulations of the effect of interactors on population growth over time (i.e., an *integrated* reproductive effect). This simulation model of huckleberry also allowed us to evaluate the contribution of seed dispersal from the survivors to huckleberry colonization (i.e., local control).

#### Population model

To examine the impact of consumers and mutualists during primary succession (Question 2) and the combined effects of species interactions and local control of propagule influx (Question 3), we developed a population model. The life cycle of black huckleberry (Fig. 1) was represented with the following 4×4 annual transition matrix model [41] (from June (*t*) when the adults flower till June the following year (*t*+1)):
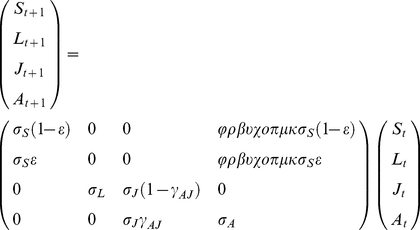
(1)in which the four stages were seed (S), seedling (L; <4 cm tall, not reproductive), juvenile (J; >4 cm tall, not reproductive), and adult (A; reproductive). The reproductive transition from adults to either seeds or seedlings is modeled as a product of reproductive rates that represent consecutive processes affecting sexual reproduction (Table 1). The seedling-to-juvenile transition probability (σ*_L_*) is likely to be extremely low. In 4 experiments over 12 years that placed scats containing huckleberry seeds in primary succession, few seedlings were produced and most of these eventually died (Bishop et al. unpublished data). Juveniles, on the other hand, had a much higher survivorship. Using this information, as well as demographic data from other long-lived shrubs [42], we set annual seed survival in seed bank (σ*_S_* = 0.05), seedling survival (σ*_L_* = 0.05), juvenile survival (σ*_J_* = 0.90) and juvenile-to-adult growth (γ*_AJ_* = 0.01) rates. We then adjusted adult survival (σ*_A_* = 0.993) and seedling establishment rate (in spring, ε = 0.10) such that the simulated population sizes after 20 years approximated the adult and juvenile population sizes observed in 2005. Huckleberry is a long-lived species where long-term experiments are difficult and demographic data are lacking. Because the estimates of survival and growth rates are approximations based on other studies (except juvenile survival), we consider the projected population growth rates most informative for evaluating the relative effects of species interactions on huckleberry population dynamics. To investigate the validity of this approach, we used sensitivity analyses for each of the six unmeasured reproductive rates (ε, σ*_S_*, σ*_L_*, σ*_J_*, σ*_A_* and γ*_AJ_*) to demonstrate the robustness of the results over a wide range of rates. The relative effect of species interactions was indeed independent of this wide range of values (Appendix S1, Figure S1).

**Table 1:** Reproductive vital rates for primary (PS) and secondary (SS) successional huckleberry at Mount St. Helens.

	Reproductive vital rate	PS	SS	Year
ϕ	number of potential berries per adult	-	424.389	2003
		778.974	209.204	2004
		569.078	215.000	2005
ρ	proportion of flowers actually produced after grasshopper herbivory	0.000	1.000	2002
		1.000	1.000	2003
		1.000	1.000	2004
		1.000	1.000	2005
β	realized (due to pollination) proportion of potential number of berries	-	1.000	2003
		0.802	1.000	2004
υ	proportion of berries not affected by fungal infection	0.994	0.895	2004
		0.999	0.805	2005
χ	proportion of uninfected berries surviving pre-dispersal predation	0.875	0.991	2004
		0.925	0.975	2005
o	potential number of seeds per surviving berry	44.911	15.479	2004
π	realized (due to pollination) proportion of potential number of seeds	0.866	1.000	2004
μ	proportion of seeds surviving post-dispersal predation	0.166	0.474	2005
κ	proportion of seeds that are viable (post-dispersal germination)	0.482	0.482	2005

Note: The last column gives the year in which the data were collected.

#### Stochastic simulation

To evaluate the potential impact of consumers and mutualists on formation of a primary successional huckleberry population, we used stochastic simulations. We used the transition matrix models to reconstruct the 20 years of colonization between 1985 and 2005. The simulations were initiated with 25 adults (the approximate number of observed survivors) and run over 20 annual time steps. Colonists (offspring of survivors or of distant, secondary successional adults) are likely to experience the same aboveground species interactions as the survivors, so we used the same survivor vital rates for colonists that survive to the adult stage. Stochasticity due to temporal variation in the interactions was included in each vital rate. For further details on the simulation procedures, see Appendix S1.

In the first set of simulations we explored the relative effect of consumer and mutualist interactions on huckleberry colonization (Question 2). In this simulation set, we compared our matrix containing PS vital rates to matrices in which no losses occur due to lack of mutualists (no pollinator limitation, i.e. the realized proportion of berries β and seeds π both set to 1), or in which no antagonistic interactions occur (i.e. no grasshoppers, fungi or pre- and post-dispersal seed predators, i.e., ρ, υ, χ, and μ all set to 1), or both. We ran these scenarios over a range of seedling survival rates (σ_L_) to ask whether the impact of the species interactions changes as ameliorating abiotic conditions enhance seedling survival. To ensure that the ranking of importance of each biotic interaction is unaffected by our estimates of the other life stages, we conducted similar simulations where we used a range of values for vital rates that were estimated from the literature (see Appendix S1, Figure S1).

In the second set of simulations we investigated the role of i) seeds from surviving adult plants compared to seeds arriving from secondary successional sources, and ii) the differences in the set of interactors between the two population types on sexual reproduction (Question 3), in a fully factorial set of scenarios. We examined the impact of these factors on colonization success (i.e. the number of adults and juveniles after 20 years). The simulations were started with either 0 or 25 adults to represent absence or presence of surviving huckleberry plants on the Pumice Plain. We evaluated the importance of long-distance seed dispersal by varying the number of seeds dispersed via coyote scats (containing 5,000 seeds each) in the primary successional area from 0 to 20 scats per year. We also simulated colonization with the species interactions resembling the biotic conditions of SS rather than those of PS. This latter simulation allowed us to see how important the differences between these two sites really are for the colonization process.

## Results

### Effect of Consumer and Mutualist Interactions on Reproduction

Overall, consumers and mutualists had a substantial effect on the reproduction of huckleberry in both PS and SS. Here, we highlight the key results of the statistical analyses (see Table S1 of full statistical analyses).

#### Pollination (β and π)

**Figure 2:**
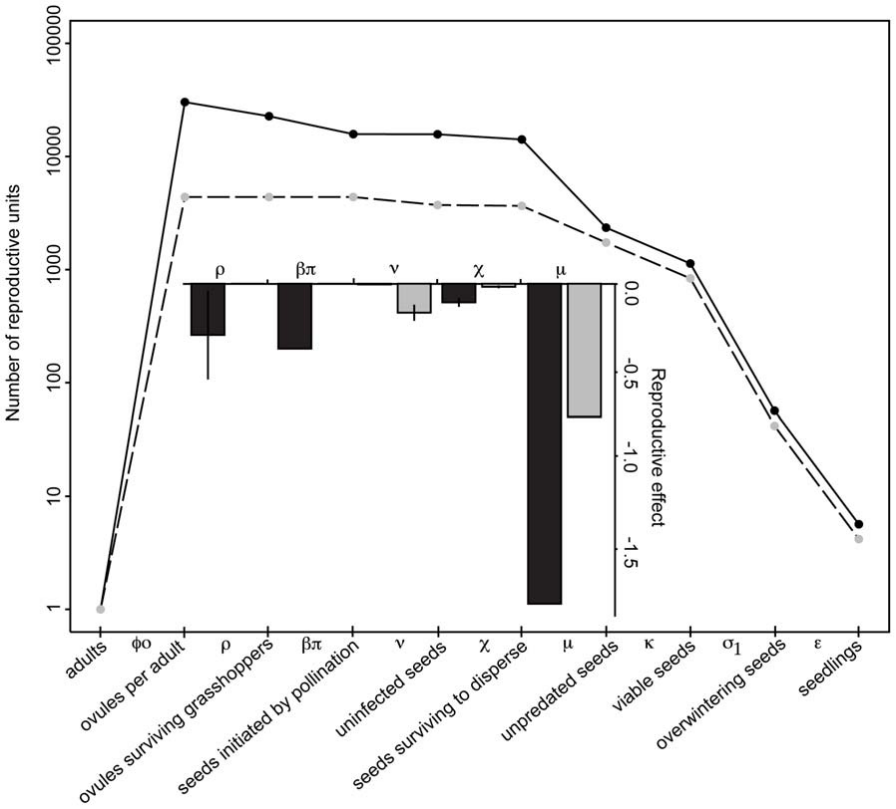
The effects of consumers and mutualists differ between primary and secondary successional huckleberry. In the primary successional population (PS), herbivory (ρ), lack of pollination (βπ), and post-dispersal seed predation (μ) reduced the number of seeds the most, which corresponded to strongly negative reproductive effects (inset: log response ratio, interannual mean and standard error; PS, black bars; SS, gray bars). The production and fate of huckleberry seeds start with one flowering adult plant and end with 5.7 primary (PS, solid line) and 4.2 secondary (SS, dashed line) successional huckleberry seedlings one year later. The Greek letters denote the transition rates between the consecutive steps (see Table 1), with the biotic interactions marked with asterisks (*). Overwinter seed survival (σ_S_) and seedling establishment (ε) rates were estimated from the literature (see Methods).

In 2003, grasshopper herbivory prevented fruit development in our PS pollination experiments. In 2004, individuals in the primary successional population (PS) had a 23% increase in fruit set with pollen supplementation, whereas there was no increase in the secondary successional population (SS) (PS natural fruit set per plant: 0.74±0.02 s.e.; PS supplemented: 0.91±0.01 s.e.; SS natural: 0.7±0.15 s.e.; SS supplemented: 0.67±0.15 s.e.). No significant main difference was found between populations (z = −1.12, p = 0.26), but supplementation (z = 2.79, p = 0.005) and population × supplement interaction (z = −2.27, p = 0.023) effects on the number of potential berries (ϕ) that developed into berries were significant. Supplementation also resulted in a 30% increase in seeds per berry in PS (44.9±13.0 s.e. vs. 34.5±7.9 s.e. in unsupplemented controls), but not in SS (15.5±2.8 s.e. vs. 15.9±8.2 s.e. in the controls). The number of seeds per berry was significantly different between populations (t = −2.36, p = 0.029), but not between treatments (t = 0.66, p = 0.519; quasipoisson error distribution and without the non-significant interaction term). The combined effect of pollination was that 31% of the potential ovules produced a seed in PS and 100% in SS (see Table 1 for the calculated values of β and π), amounting to reproductive effects of −0.365 for PS and 0 for SS (Fig. 2 inset).

#### Fungal infection (υ) and seed predation (χ)

The proportion of infected fruit, and thus seed death attributable to fungal infection, was 39 times higher in SS (0.150) than in PS (0.0038) (z = 2.81, p = 0.005), which equates to log response ratios of −0.163 and −0.004 respectively (Fig. 2 inset). Whereas this proportion was constantly low in PS, it changed in SS from 0.132 in 2004 to 0.195 in 2005 (year effect: z = 1.55, p = 0.12; interaction effect: z = −2.81, p = 0.005). Populations also differed in rates of pre-dispersal seed predation (z = 5.32, p<0.001): a lower proportion of fruits were damaged in SS (2004: 0.0089±0.0033 s.e.; 2005: 0.025±0.012 s.e., reproductive effect: 0.017) than in PS (2004: 0.125±0.060 s.e.; 2005: 0.075±0.013 s.e., reproductive effect: 0.105) (interaction effect: z = −5.32, p<0.001).

#### Post-dispersal seed predation (μ)

The 2004 eruption of Mount St. Helens prevented us from surveying seed predation plots in September 2004. In plots established in August 2005, we found that significantly more seeds disappeared from PS (reproductive effect: −1.796) than from SS plots (reproductive effect: −0.747, Figure 2 inset; t = 2.192, p = 0.035).

### Question 1: Do Species Interactions Vary with Successional Context?

#### Combined effects

Reproductive effects were strikingly different between PS and SS (Fig. 2 inset). On average, antagonistic interactions had a much stronger negative effect than the lack of pollination in PS. For example, within PS, the reduction in number of seeds due to insufficient pollination (βπ) was less than the combined reductions due to grasshopper herbivory (ρ), predispersal seed predation (χ), and especially post-dispersal seed predation (μ). Overall, the negative effects of the PS community are considerably stronger than those of the SS community.

**Figure 3:**
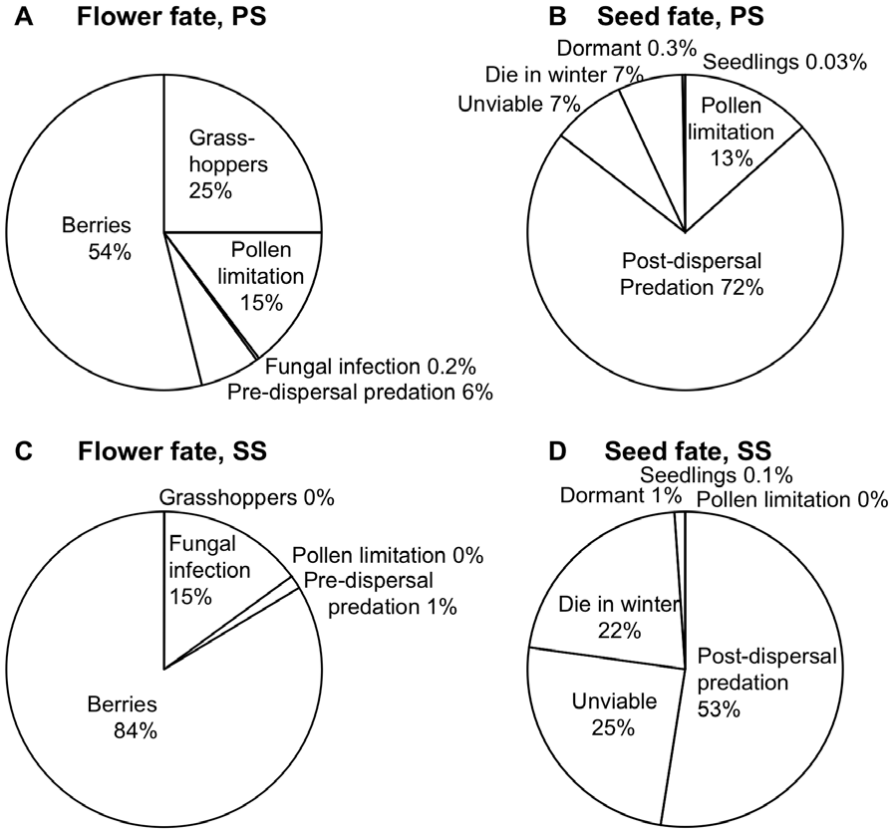
Fates of primary and secondary successional huckleberry. Pie charts show the fates of potential flowers (A,C) and potential seeds (B, D) in PS (A, B) and SS (C, D). All flowers that do not develop into dispersing berries are lost due to either to grasshoppers, insufficient pollination, fungal infections or pre-dispersal predation. All potential seeds that do not establish as seedlings are lost due to herbivory, insufficient pollination, post-dispersal predation, unviability or over-winter mortality, or remain dormant in the seed bank.

A greater proportion of flowers per plant developed into fruit available for dispersal in SS (Fig. 3B, 3D). This was attributable to the much greater effect of grasshopper damage and pollen limitation in PS. However, the average number of fruits and seeds per plant after pre-dispersal seed predation was actually much higher in PS, partially due to the 3× higher fecundity (Fig. 2). Surprisingly, this nearly 4.5-fold difference in seeds per plant (16,300 vs. 3,660) was largely eliminated by higher post-dispersal seed predation in PS (Figs. 2, 3A, 3C). The effects of more strongly negative interactions (ρ, β, χ, π and μ) in PS appear to be balanced by the positive contributions of increased flower (φ) and seed (ο) production (Fig. 2), perhaps due to reduced competition on relictual pre-eruption soils in this low-density population.

### Question 2: What are the Impacts of Consumers and Mutualists during Primary Succession?

**Figure 4:**
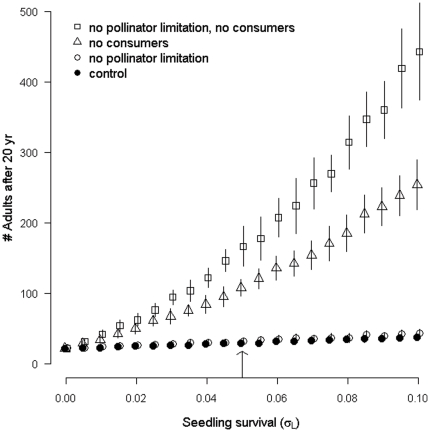
The integrated reproductive effect of consumers was greater than that of mutualists. Mean (±s.d.) number of adults on the primary successional Pumice Plain after simulating the 1985–2005 period as a function of seedling survival (σ*_L_*) using a stochastic model (see Methods section). All simulations started with 25 survivors, used the PS species vital rates, and had 10 coyote scats arriving annually from the nearby secondary successional population. The four scenarios were: ‘control’ = all species interactions as observed, ‘no pollinator limitation’ = no reduced berry (β = 1) or seed (π = 1) production due to insufficient pollination, ‘no consumers’ = no losses due to grasshoppers (ρ = 1), fungal infections (υ = 1), pre-dispersal predation (χ = 1) or post-dispersal predation (μ = 1), and ‘no pollinator limitation, no consumers’ = neither losses due to insufficient pollination nor due to antagonists. The arrow denotes our basic scenario for which plant survival (e.g. σ*_L_* = 0.05) and growth rates have been set to match the observed trends in adults and juveniles over the 1985–2005 period.

The projected population growth rate (λ) was higher for PS than for SS (1.04 vs. 1.00; without external seed sources). For the stochastic simulation, removing antagonists resulted in far more additional adult recruitment than increasing pollinator services (“no pollinator limitation”; Fig. 4). Furthermore, our simulations found that increasing pollinator services did not increase the number of adults unless the antagonists were absent. This non-additive statistical interaction becomes increasingly important when the population is rapidly growing (i.e. at higher seedling survival rates). Thus, our simulation revealed a more complex relationship among the consumers and mutualist effects than demonstrated by the log response ratio estimate of reproductive effect, which only identified that consumer pressure is on average more limiting than the lack of mutualists.

**Figure 5:**
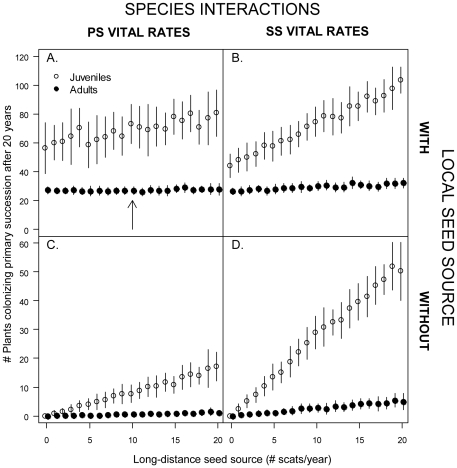
The number of adults and juveniles on the primary successional Pumice Plain after simulating 20 years of colonization. Simulations were started with either 25 flowering plants as a source of local seeds (long-distance and local source: A,B) or 0 adults (long-distance source only: C,D). The stochastic population model (see Methods section) reflected either community interactions in the primary (A,C) or secondary (B,D) successional habitat. The number of coyote scats (containing 5,000 seeds each) arriving on the Pumice Plain from the nearby secondary successional population (long-distance seed source) are varied on the x-axis. The arrow denotes our basic scenario for which plant survival and growth rates have been set to match the observed trends in adults and juveniles over the 1985–2005 period.

Sensitivity analyses also revealed that the magnitude of the effects of consumers and mutualists are highly dependent upon values of survival and growth parameters. However, the relative effects of consumers and mutualists are independent of a wide range of estimates for the six unmeasured vital rates (Fig. 5, also see Figure S1). Regardless of the value of the vital rate, supplementing pollination (“no pollinator limitation”) had a minimal effect on adult recruitment in contrast to the relatively large positive effect of removing consumer interactions (“no consumers”). In addition, sensitivity analyses indicated that the effects of “no consumers” and “no pollinator limitation and no consumers” would become increasingly larger as survival and growth increased (Fig. 4, see Figure S1). Thus, it is likely that our estimates of the six unmeasured vital rates do not bias the relative effects of the consumer and mutualist interactions on PS adult recruitment in our simulation.

### Question 3: What are the Effects of Local Control of Propagule Influx?

Local seed dispersal from surviving adults within PS resulted in more colonization than without local dispersal, confirming that local dispersal is an important factor that compensates for losses from strong consumer pressure during primary succession (Fig. 5A, 5B vs. 5C, 5D). Even if a set of interactors is able to slow the rise of a particular species to dominance [15], a relatively small amount of local control allows for a more rapid increase. In addition, colonization was greater when we assumed an interaction set typical of SS than when we used a set from PS (Fig. 5B, 5D vs. 5A,C). We attribute this latter pattern to the lower antagonistic vital rates in SS (see also Fig. 2), especially the lower post-dispersal seed predation rate, that caused more juveniles to recruit per scat arriving from the external seed source. Simulated huckleberry population growth was much more rapid under the set of SS vital rates.

## Discussion

Our study finds that the effect of multiple species interactions on huckleberry depends on successional context, and provides support for the importance of biotic interactions during primary succession. The combined negative effect of consumers and insufficient pollination was greater for primary successional huckleberries (PS) than for secondary successional huckleberries (SS), and furthermore, these interactions have unusually large effects on PS populations. Our simulation models indicate that under primary successional conditions, the resulting seed loss should substantially diminish growth of the colonizing population. Our simulations also show that the dynamics of the colonizing population in primary successional habitat are strongly dependent on seed dispersal from both local survivors and the secondary successional population, counteracting seed loss and allowing for more rapid colonization.

### The effect of consumers and mutualists in succession

The considerable difference between PS and SS may be attributed to pollinator and consumer behavioral responses to the sparse spatial distribution of huckleberry plants and other resources in PS compared to SS. The lack of effective pollination may be due to a shortage of pollinators, combined with the effect of geitonogamous or interspecific pollen transfer that often accompanies low plant density [e.g.], [ 43,44]. It is worth mentioning that a complete absence of pollinators would result in no huckleberry reproduction, and no population growth without dispersal from external sources (e.g., no pollinators in Fig. 4 would produce a horizontal line), because huckleberry is largely self-incompatible.

The greater risk of herbivory and seed predation to primary successional plants may have several related causes. Isolated plants may each have a larger “basin of attraction” for foraging insect seed predators. Rather than searching the surrounding primary successional landscape for scarce food sources, once a forager has located an isolated plant, it is likely to stay, possibly resulting in more consumers per plant [e.g., 45]. Insect consumers in primary successional areas are also more likely to escape their predators and thus reach higher population densities [e.g., 46]. Resource density in many areas of the Pumice Plain appears too low to support insectivorous vertebrate predators, such as birds and most small mammals, and low-density areas are depauperate in arthropod predators [29], [47], [48].

Our comparisons of simulated population growth demonstrated that insufficient pollination and reduced survival due to consumers combine to greatly limit population growth in PS. This result depends critically on the rate of seedling survival - at very low seedling survival (σ*_L_*<0.01), the number of seeds lost does not matter, while at higher seedling survival rates, removing the consumer interactions and increasing pollination rapidly increases recruitment to the adult class (Fig. 4, “no pollinator limitation, no consumers”). Releasing the population from consumer pressure, without supplementing pollination, allows for a somewhat less rapid increase in recruitment to the adult class as seedling survival increases (Fig. 4, “no consumers”). On the other hand, an increase in seedling survival does not appreciably increase population growth in the presence of consumers (“no pollinator limitation” and “control”). In other words, there is little effect of increasing pollination on population growth except in the absence of consumers. Thus, our simulation provides strong evidence that consumers, much more so than a shortage of mutualists, retard colonization of important later successional species at Mount St. Helens, and may therefore impact community trajectories. Vertebrate herbivores, such as moose and hares, can accelerate rates of primary succession in boreal flood plains and deglaciated sites [49], [50]. In contrast, there are only a few examples of invertebrate herbivores impacting primary succession [16], [47]. One of the best-documented cases is also from the Mount St. Helens Pumice Plain, where specialist insect herbivores decrease population growth rate and the rate of spread of alpine lupine (*Lupinus lepidus*) [17], [18], [47], which decelerates succession at large spatial scales, but temporarily accelerates it at small scales by releasing lupine-held resources [51], [52], [53].

### Succession and dispersal

The strong effect of consumers within primary successional communities could be a general mechanism that contributes to slower species accumulation during primary succession compared to secondary succession. Certainly, without the mitigating effect of local control in our primary successional population, colonization would be extremely slow (Fig. 5). The presence of biological legacies (remnant organisms and associated structures, living or dead) within a disturbed landscape is thought to be a major factor facilitating ecosystem recovery, especially after very large-scale disturbances [54], [55]. Indeed, our simulations (and genetic analyses; [34]) demonstrated a significant demographic impact of even a small number of survivors on the rate of colonization. Our results are in contrast to other studies, based on vegetation surveys of secondary successional refugia and surrounding primary successional sites at Mount St. Helens, which concluded that refugia facilitated invasion in the immediately adjacent areas of primary succession by vagile, pioneering species, but not by later successional species such as huckleberry [26], [56]. These contrasting conclusions likely stem from the behavior of fruit-dispersing animals in primary successional habitat: frugivores that leave refugia do not linger in adjacent bare areas and thus seeds are dispersed long distances and deposited at low densities. Overall, we highlight the need for more studies of community assembly that include both successional stages, investigate the relative influence of local vs. donor control, and attempt to disentangle biotic and abiotic factors.

Using matrix model simulations, we were able to explore how alternate sets of interactors and dispersal interact to influence rates of colonization. But, like many models, incomplete information limits our ability to make broader conclusions with our simulation model of colonization. As mentioned, the magnitude (but not relative importance) of species interaction effects on population growth rates depends on the level of recruitment (Fig. 4), though the sparse recruitment rates that we have observed previously suggest that our estimated value is reasonable. In addition, the seed-to-seedling transition rate may not be constant over time, and may actually differ between PS and SS. Similarly, population growth rates will depend on other stages of the life cycle, though sensitivity analyses of these other life stages (see Figure S1) showed that our estimates do not affect conclusions regarding the relative impact of the interactions we focused on in this study. Although the population dynamics of woody plant species are not typically sensitive to early life stages [57], our results suggest that seedling survival may actually be important for woody plants in nonequilibrium systems with temporal variation in the strength of consumer interactions.

### Biotic interactions and successional context

We found that in primary succession the limiting effects of mutualists and consumers on colonizing plants were not only severe, but also amplified relative to their effect in more mature secondary succession. Our results also indicate that these reproductive effects can translate to substantial effects on population growth (Figs. 4 and 5), and thus can be interpreted as interaction strengths (the effect of one species on the population growth of another, [58]). The distribution of interaction strengths in early successional communities, and how that distribution is likely to change through successional time, are virtually unstudied. Empirical estimates from “non-successional” trophic webs repeatedly reveal a distribution characterized by a few strong interactions and many weak ones [25], [58], and models of consumer-resource interaction indicate that this skewed distribution confers stability [59], [60], [61]. Although not intended as models of succession (except [61]), these models, together with empirical studies such as ours that document the occurrence of strong interactions affecting early plant colonists, suggest that successional communities in the early stages of assembly may exhibit more strong interactions than in later successional communities, or that they may lack other stabilizing properties that only occur in more diverse or successionally-advanced communities, such as nestedness [62]. The instability of young communities may produce radical shifts in the population dynamics of the existing interactors, which may directly change the distribution of interaction strengths via predator-prey time lags, extinction of predator or prey, or predator switching to more abundant prey. Alternatively, an unstable interaction strength distribution may continue until other species colonize and buffer the existing strong interactions with weaker interactions. For both of these scenarios, community assembly should continue if the unstable distribution of interaction strengths drives successional change. This new perspective on community succession calls for more studies that investigate the successional dependence of interaction strengths.

Our study reveals an alternative view of succession, in which the distribution of interaction strengths shifts from a relatively high proportion of strong interactions toward distributions that are characteristic of stable communities. In particular, the combined effect of strong consumer pressure and absence of mutualists can significantly slow plant colonization, but this effect may be offset by local control of propagule influx. Although particular interactors may vary among systems, our study concludes that it is critical to consider the dependence of interaction strengths on successional context, and the potential importance of consumer and mutualist interactions for terrestrial primary succession.

## Supporting Information

Appendix S1 **Stochastic simulation details.** Details of the stochastic simulation used to investigate the effect of consumers and mutualists on huckleberry colonization of primary successional habitat.
(DOC)

Figure S1 **The relative effect of species interactions was independent of the unmeasured reproductive rates.** Mean (±s.d.) number of adults on the primary successional Pumice Plain after simulating the 1985–2005 period as a function of seed survival (σ*_S_*), establishment (ε), seedling survival (σ*_L_*), juvenile survival (σ*_J_*), juvenile to adult growth (γ*_AJ_*), and adult survival (σ*_A_*) using a stochastic model. The arrow denotes our basic scenario for which plant survival (e.g. σ*_L_* = 0.05) and growth rates have been set to match the observed trends in adults and juveniles over the 1985–2005 period.
(TIF)

Table S1 **Effect of species interactions on huckleberry reproduction.** Full statistical analysis of species interactions on huckleberry reproduction.
(DOC)
