# Supplementary material for: The Effect of Consumers and Mutualists of Vaccinium membranaceum at Mount St. Helens: Dependence on Successional Context
Source: PLoS One. 2011 Oct 20;6(10):e26094. doi: 10.1371/journal.pone.0026094 (PMC3197599; doi:10.1371/journal.pone.0026094)
Supplement: Appendix S1 — Stochastic simulation details. Details of the stochastic simulation used to investigate the effect of consumers and mutualists on huckleberry colonization of primary successional habitat. (DOC) [file pone.0026094.s001.doc]

**Supporting Information**

**Stochastic simulation details**

We used stochastic simulations of our transition matrix-based population model to investigate the effect of consumers and mutualists on huckleberry colonization of primary successional habitat. The life cycle of black huckleberry (Fig. 1) was represented with the following 4x4 annual (from June (*t*) when the adults flower till June the following year (*t+*1)) transition matrix:

1

in which the four stages were seed (S), seedling (L; <4 cm tall, not reproductive), juvenile (J; >4 cm tall, not reproductive), and adult (A; reproductive). The reproductive transition from adults to either seeds or seedlings is modeled as a product of vital rates that represent consecutive processes affecting sexual reproduction (Table 1).

In the stochastic simulations, negative binomial distributions were used to draw random numbers to determine how many flowers (φ) each adult could potentially make, and also to determine the potential number of seeds (ο) of each berry (see below for the shapes of these distributions). To simulate the fate of each potential berry and potential seed, we used binomial distributions to stochastically simulate survival (or death) at each step in the reproductive pathway (ρ, β, υ, χ, π, μ, κ and ε). Binomial distributions were also used to simulate the survival (σ) and growth (γ*_AJ_*) of individual plants. The binomial distributions were shaped by the observed mean for a vital rate in a certain year (see Table 1 and Methods section in the main text for the vital rate means).

Whenever more than one year of data was available we first randomly drew one of those years at each time step (all years had the same probability of being chosen each time), and then used the observed mean from that year for random number generation for that vital rate. We used this procedure because we did not have full data sets for different years to draw entire data sets of individual years, and because we think that by using these binomial and negative binomial distributions we are more accurately mimicking natural stochasticity.

The negative binomial distributions were parameterized by both the mean and the variance of the data: respectively, 778.97 and 25296.62 for potential berry numbers in PS in 2004, 569.08 and 80408.55 for potential berry number in PS in 2005, 424.39 and 88363.67 for potential berry number in SS in 2003, 209.20 and 13747.00 for potential berry number in SS in 2004, 215.00 and 13455.56 for potential berry number in SS in 2005, 44.91 and 1193.33 for potential seed number in PS in 2004, and 15.48 and 32.72 for potential seed number in SS in 2004.

Using stochastic simulations, we examined the sensitivity of biotic interaction effects to our estimates of the unmeasured vital rates: seed survival (σ*_S_*), establishment (ε) (note that σ*_S_* and ε together determine the fate of seeds in the seed bank), seedling survival (σ*_L_*), juvenile survival (σ*_J_*), juvenile to adult growth (γ*_AJ_*), and adult survival (σ*_A_*). We conducted simulations where we used a range of values for each unmeasured vital rate. All simulations started with 25 survivors, used the primary succession species interactions, and had 10 coyote scats arriving annually from nearby secondary succession populations. The four scenarios were: ‘control’ = all observed species interactions, ‘no pollinator limitation’ = no reduced berry (β=1) or seed (π=1) production due to insufficient pollination, ‘no consumers’ = no losses due to grasshoppers (ρ=1), fungal infections (υ=1), pre-dispersal predation (χ=1) or post-dispersal predation (μ=1), and ‘no pollinator limitation, no consumers’ = neither losses due to insufficient pollination nor due to antagonists.

Our sensitivity analysis showed that although the magnitude of the effects of biotic interactions on PS adult recruitment varied with values of the six estimated survival and growth parameters, the relative effects of these interactions were independent of these parameters (Fig. S1). Biotic interactions are of little influence when survivorship and growth were low (as when abiotic limitation is severe), but of increasing importance for adult recruitment as survival and growth increased. However, regardless of whether survival and growth were low or high, increasing pollinator services (“no pollinator limitation”) had a very small effect on adult recruitment compared to the large positive effect of removing consumer interactions (“no consumers”) and increasing pollinator services and removing consumer interactions (“no pollinator limitation, no consumers”) (Fig. S1). Therefore, it appears that the relative effects of the biotic interactions are robust to our estimates of the six unmeasured vital rates.

**Figure S1.** **The relative effect of species interactions was independent of the unmeasured reproductive rates.** Mean (±s.d.) number of adults on the primary successional Pumice Plain after simulating the 1985-2005 period as a function of seed survival (σ*_S_*), establishment (ε), seedling survival (σ*_L_*), juvenile survival (σ*_J_*), juvenile to adult growth (γ*_AJ_*), and adult survival (σ*_A_*) using a stochastic model. The arrow denotes our basic scenario for which plant survival (e.g. σ*_L_*=0.05) and growth rates have been set to match the observed trends in adults and juveniles over the 1985-2005 period.
