# Supplementary figures and images for: The Effect of Consumers and Mutualists of Vaccinium membranaceum at Mount St. Helens: Dependence on Successional Context
Source: PLoS One. 2011 Oct 20;6(10):e26094. doi: 10.1371/journal.pone.0026094 (PMC3197599; doi:10.1371/journal.pone.0026094)

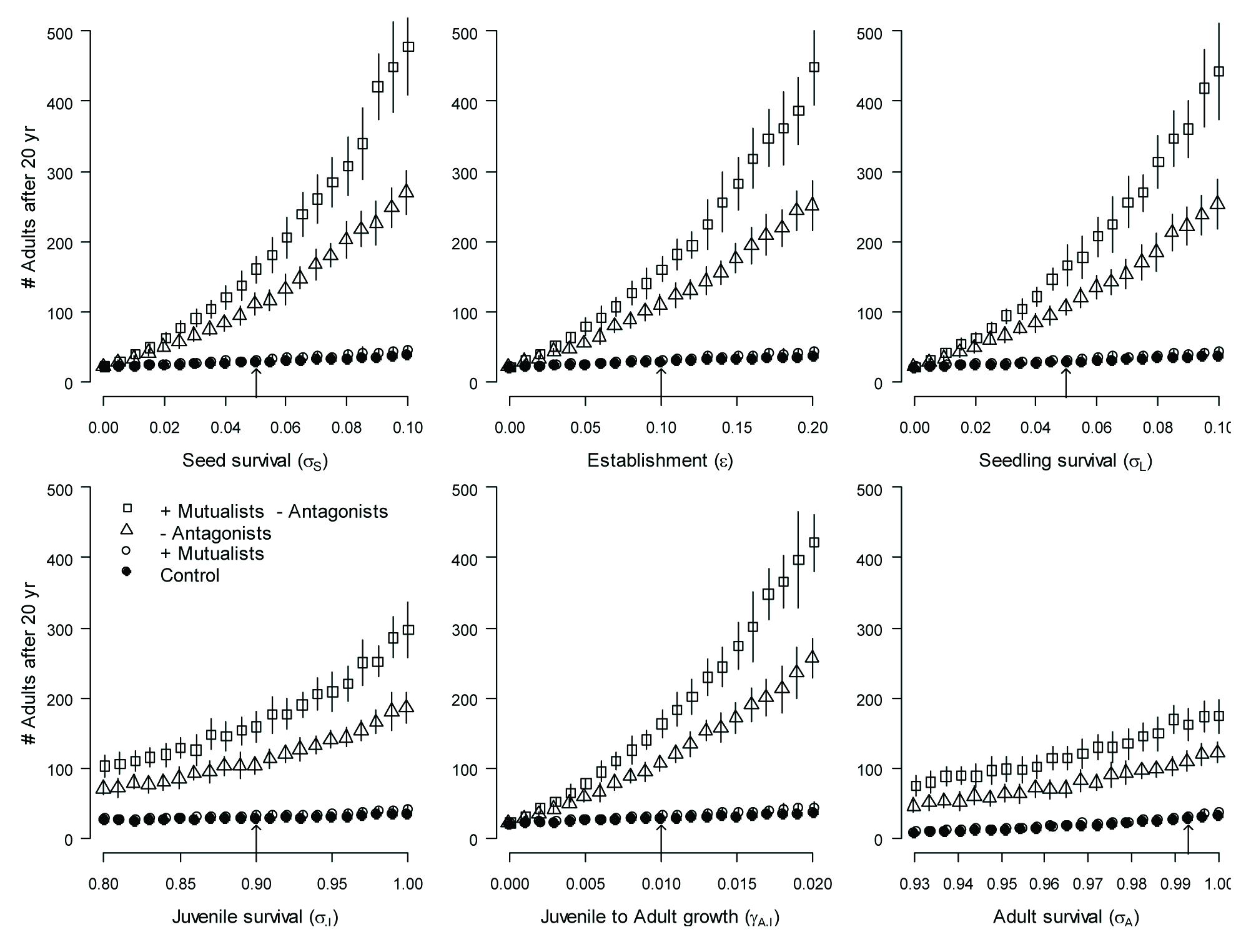

Supplement: Figure S1 — The relative effect of species interactions was independent of the unmeasured reproductive rates. Mean (±s.d.) number of adults on the primary successional Pumice Plain after simulating the 1985–2005 period as a function of seed survival (σS), establishment (ε), seedling survival (σL), juvenile survival (σJ), juvenile to adult growth (γAJ), and adult survival (σA) using a stochastic model. The arrow denotes our basic scenario for which plant survival (e.g. σL = 0.05) and growth rates have been set to match the observed trends in adults and juveniles over the 1985–2005 period. (TIF) [file pone.0026094.s002.tif]
