# Supplementary material for: The Effect of Consumers and Mutualists of Vaccinium membranaceum at Mount St. Helens: Dependence on Successional Context
Source: PLoS One. 2011 Oct 20;6(10):e26094. doi: 10.1371/journal.pone.0026094 (PMC3197599; doi:10.1371/journal.pone.0026094)
Supplement: Table S1 — Effect of species interactions on huckleberry reproduction. Full statistical analysis of species interactions on huckleberry reproduction. (DOC) [file pone.0026094.s003.doc]

**Supporting Information**

**Effect of species interactions on huckleberry reproduction**

**Table S1.** Full statistical analysis of species interactions on huckleberry reproduction.

| Interaction effect | Vital rate | Parameter | Estimate ±s.e. | Statistic | p-value |  |
| --- | --- | --- | --- | --- | --- | --- |
| Fruit set due to pollination | β | Intercept (PS & Natural) | 1.033 ±0.254 | z = 4.065 | <0.0001 | *** |
|  |  | Secondary succession | -0.414 ±0.303 | z = -1.365 | 0.1724 |  |
|  |  | Supplemental pollination | 1.312 ±0.470 | z = 2.789 | 0.0053 | ** |
|  |  | Secondary × Supplemental | -1.366 ±0.525 | z = -2.602 | 0.0093 | ** |
| Seed set due to pollination | π | Intercept (PS & Natural) | 3.634 ±0.129 | t = 28.195 | <0.0001 | *** |
|  |  | Secondary succession | -0.952 ±0.284 | t = -3.356 | 0.0012 | ** |
|  |  | Supplemental pollination | 0.399 ±0.162 | t = 2.467 | 0.0155 | * |
| Infection by fungus | υ  | Intercept (PS & 2004) | 6.462 ±1.317 | t = 4.906 | <0.0001 | *** |
|  |  | Secondary succession | -4.166 ±1.308 | t = -3.186 | 0.0025 | ** |
|  |  | Year 2005 | -0.943 ±0.460 | t = -2.048 | 0.0457 | * |
| Pre-dispersal predation | χ | Intercept (PS & 2004) | 2.019 ±0.302 | t = 6.685 | <0.0001 | *** |
|  |  | Secondary succession | 2.397 ±0.876 | t = 2.737 | 0.0085 | ** |
|  |  | Year 2005 | 0.584 ±0.592 | t = 1.103 | 0.2751 |  |
| Post-dispersal predation | μ | Intercept (PS) | -1.992 ±0.336 | t = -5.931 | <0.0001 | *** |
|  |  | Secondary succession | 0.988 ±0.451 | t = 2.192 | 0.0353 | * |

Generalized linear regression models were used to estimate the effect sizes of succession type, treatment and year, where applicable. Non-significant interactions between these factors were removed. All analyses had binomial error structures, except for the π model (poisson). The error structures were adjusted (with the ‘quasibinomial’ and ‘quasipoisson’ commands in R) when the dispersion parameter was high: 24 for π, 23 for χ, 18 for υ and 194 for μ.
